# Nurturing comfort: qualitative exploration of pain management techniques among urban teaching hospital nurses

**DOI:** 10.1097/PR9.0000000000001325

**Published:** 2025-08-20

**Authors:** Awube Menlah, Evans Appiah Osei, Gloria Asomani, Isabella Garti, Dorothy Baffour Awuah, Thomas Kwame Asante Tata, Stella Appiah

**Affiliations:** a Charles Darwin University to School of Nursing and Midwifery, Valley View University; b Purdue University, West Lafayette, IN, United States; c Registered Nurse, Ascension St. Agnes, Baltimore, Maryland USA; d School of Nursing and Midwifery, Valley View University, Accra, Ghana; e 37 Millitary Hospital, Accra, Ghana; f Nursing Department, School of Nursing and Midwifery, Valley View University, Accra, Ghana

**Keywords:** Comfort, Pain management, Paediatric, Nurses, Management

## Abstract

**Introduction:**

Despite advances in pain assessment and management, pediatric pain remains undertreated, posing a significant challenge including mortalities. This hinders effective pain management for children, often due to difficulties in pediatric pain assessment by nurses. This study explores pain management practices among pediatric nurses at Korle-Bu Teaching Hospital, Accra.

**Methods:**

A qualitative descriptive design was used in this study to select 21 practicing nurses using purposive sampling. A semi-structured interview guide was used to collect data via face-face interviews, and data were analysed using inductive content analysis.

**Results:**

Four major themes and 8 subthemes emerged from the findings. Pediatric pain assessment was primarily guided by the experience of care providers rather than routine practices, with children themselves serving as the main indicators of pain rather than relying on parents. Despite various barriers, health care providers prioritized pediatric pain management by integrating compassion, flexibility, and cultural sensitivity into their approach.

**Conclusion:**

Pediatric nurses were attentive and provided prompt pain relief but faced challenges with pain assessment and medication administration, particularly with suppositories. Implementing standardized protocols for pain management and providing resources to support nurses in overcoming these challenges can improve the quality of care provided to paediatric patients.

## Abstract 2

This paper adds the identification of the unique challenges faced by nurses and description of strategies in managing pediatric pain in a middle-income country.

## 1. Introduction

Despite significant advancements in pain assessment and management, addressing pediatric pain effectively remains a considerable challenge.^4,28^ This complexity poses substantial difficulties for nurses tasked with assessing pediatric pain, often leading to its undertreatment.^7,8^ Pediatric pain is a critical issue associated with increased mortality and morbidity rates, as well as considerable distress among parents and nurses.^1,25^ Moreover, there are consistent reports that children's pain is frequently ignored or underestimated.^3,22^

Children experience a wide range of pain due to various conditions, including vaso-occlusive crises, physical trauma, and procedures such as surgeries.^2,18,21^ Common areas of pain experienced by children include the legs (26.5%), abdomen (16.6%), head/neck (16.6%), and back (14.2%).^17,19,25^ Poor management of this pain affects children's physical, psychological, and social well-being, underscoring the need for better pain assessment and intervention strategies.

Recent research has identified several factors that contribute to suboptimal pain management in children, particularly in developing countries. These factors include high workloads and insufficient time for proper pain assessment.^1^ In low- and middle-income countries, notably in regions such as Africa, poor pediatric pain assessment and management remain prevalent, a disparity especially pronounced when compared to developed nations.^29^ This inadequacy is, in part, attributed to a lack of sufficient knowledge among nurses about pediatric pain management.^13^ Furthermore, studies involving children in West Africa are limited.^26^ A study conducted across 4 hospitals in Ghana on culturally contextual pediatric pain management found that common sources of pain for children included fractures, sickle cell disease, and skin-breaking procedures such as intramuscular injections, intravenous cannulation, lumbar puncture, surgical operations, and wound dressings, among others.^11^

In Ghana, ineffective pain management has been attributed to significant knowledge gaps and concerns regarding drug dependency.^5^ Furthermore, some authors identified deficits in the knowledge of postoperative pain management among nurses in 4 district hospitals, despite their generally positive attitudes toward pain management.^16^ Notably, there is a paucity of information regarding the experiences of pediatric nurses with pain management in Africa, and specifically in Ghana. Given the scarcity of research focused on pediatric pain management in this region, this study aims to assess the pain management practices among nurses at Korle-Bu Teaching Hospital in Accra, thereby addressing a critical gap in the existing literature.

## 2. Design and setting

The researchers used a descriptive qualitative design to examine nurses' perspectives on pain management in hospitalized children, effectively capturing the complexities of care and diverse viewpoints.^24^ The study was conducted in the pediatric unit of Korle-Bu Teaching Hospital in Accra, which has been operational since 1923, with a pediatric unit added in 1964.^9^ The nurses in this unit have managed pediatric pain for at least 1 year, with some having up to 10 years of experience, and they handle pain management on a daily basis. This unit serves as a tertiary referral center for children under 13, addressing a broad range of medical and surgical issues. The hospital handles approximately 1,500 patients daily, admits around 250, and performs about 1,000 surgeries annually, highlighting its essential role in regional pediatric care.

## 3. Sampling techniques and size

The study focused on professional nurses at Korle-Bu Teaching Hospital in Accra who had worked in pediatric units for at least 6 months. This criterion ensured the inclusion of nurses experienced in pediatric pain management with both pharmacological and nonpharmacological approaches. Nurses from 6 pediatric wards who met the criteria and consented were selected through purposive sampling.

By the 19th participant, data saturation was reached, meaning no new themes or perspectives were expected from additional interviews. Two more interviews were conducted, but the information was consistent with previous data. The final sample size was 21. Semi-structured interviews allowed for in-depth, open-ended responses. The interview schedule, developed through literature review, expert consultation, and iterative refinement, used semi-structured questions and probes to thoroughly address the research objectives.

## 4. Data collection and ethical procedure

Participants from the Pediatric Unit at Korle-Bu Teaching Hospital were selected for semi-structured, open-ended interviews, which were conducted in English, as all participants were proficient in the language. Interviews were scheduled at participants' preferred times and locations, with 18 choosing their homes or other private settings and 3 at the hospital. Each participant received a briefing on the study's purpose and rationale, provided verbal consent, and had the option to give written consent. Of the 28 nurses who initially agreed, 2 later withdrew by phone, and the remainder were informed of the study's termination due to data saturation, which they accepted. Data collection spanned 2 months, with interviews lasting 40 to 60 minutes, recorded and supplemented by field notes. Ethical clearance was granted by the Dodowa Health Research Centre Institutional Review Board (DHRCIRB) with protocol ID DHRCIRB/37/03/20, and administrative approval was secured from the hospital's medical directorate. The department heads and chief nursing officer also gave permission. After participants signed consent forms, they were assured of voluntary participation and were given pseudonyms to ensure anonymity.

## 5. Data analysis

Data collection and analysis were conducted simultaneously in this study. Interviews were transcribed verbatim in English and analyzed using inductive content analysis, a method well-suited for text-based data.^14,27^ The process involved coding and identifying patterns in the data, with transcriptions manually verified against audio recordings for accuracy. The first 3 authors conducted the initial analysis, whereas the fourth and fifth authors reviewed and validated the findings. This analysis included identifying open codes, grouping related codes into subconcepts, categories, and themes.^12^ Recurring themes were organized and coded using NVivo version 12. The researchers engaged in independent code review. The process of engaging in a dialectic process helped enrich the analysis and provided a deeper understanding of the research subject. It also helped reach consensus, where the goal is to arrive at a unified, objective understanding based on empirical evidence to validate findings and enhance reliability. Throughout, the focus was on understanding pain as a subjective and socially constructed experience, considering interactions and contexts.

## 6. Rigour

Rigorous qualitative research ensures the integrity of the findings. Key attributes for rigorous qualitative research are transparency, credibility, dependability, comparativeness, and reflexivity.^10^ We maintained rigor by strictly adhering to the study objectives.

All authors reviewed the paper before submission, and additional experts in pain and qualitative methods also conducted peer reviews. Field notes were kept to document participants' reactions, such as facial expressions, that could not be captured in recordings. Data transcriptions were done verbatim, preserving the participants' original expressions throughout the analysis. The research team, comprised of nurses and midwives, includes 2 PhD holders in nursing, each with 6 to 15 years of clinical experience in pediatric pain management. This extensive professional background ensures a thorough understanding of the subject matter, enhancing the credibility and relevance of the study's findings.

## 7. Results

### 7.1. Demographic data of participants

See Table 1 for details of patient's demographic information.

**Table 1:** Demographic data table of participants.

Variable	Frequency (n = 21)	Percentage (%)
Age		
27–30	18	85.7%
31–35	3	14.3%
Units		
Babies unit	6	28%
Pediatric emergency	4	19%
PICU	2	9.5%
Pediatric surgical	3	14%
Pediatric medical	4	19%
Pediatric oncology	2	9.5%
Years of experience		
8–12 mo	4	19%
2–5 y	9	43%
6–9 y	8	38%
Sex		
Male	6	28.5%
Female	15	71.4%
Rank		
SN	5	24%
NO	7	33.3%
SNO	3	14%
PNO	2	9.5%
SSN	4	19%
Tribe		
Ga	2	9.5%
Ewe	8	38%
Akan	8	38%
Dangbe	3	14%

Source: Interview, 2021.

### 7.2. Organisation of themes

Four major themes were generated from the data. Each theme was further divided into subthemes. See Table 2 for details of the themes and subthemes.

**Table 2:** Themes and subthemes.

Themes	Subthemes
Experienced-based pain assessment shaped by children's active communication	Children's active role in pain communication
	Pain assessment guided by experience rather than routine process
Prioritizing pain management and barriers faced	Prioritizing effective pain management
	Barriers to paediatric pain management (medication and assessment)
Blending compassion, flexibility, and cultural practices	Integrating compassion and flexibility in pain management beyond protocols
	Cultural resonance in pain relief: the impact of soothing traditions on crying
	Empowering families through pain management
Health care providers proactive strategies and sensitivity in pediatric pain management	Clinician assessment and strategies in paediatric pain management
	High sensitivity and prompt response to paediatric pain by nurses

### 7.3. Theme 1: experienced-based pain assessment shaped by children's active communication

This major theme describes the strategies used to manage pain in children. Participants indicated that they used different approaches to assess and manage pain.

#### 7.3.1. Subtheme 1: children's active role in pain communication

Participants were struck by the various ways children communicated their pain, often finding them to be surprisingly proactive in expressing discomfort. They relied on children's facial expressions, vocalizations, changes in vital signs, and verbal cues to assess pain. Crying was frequently highlighted as the primary indicator, especially in infants.

“Children differ from adults in their symptoms; often, excessive crying can signal pain in children. The intensity of their pain may be reflected in the intensity of their cries. Having work with children for several years I know which of the cries signifies pain”

(Esi, 31 years)

“Assessing pain levels is essential, and during the assessment, you can observe facial expressions and a baby's crying patterns. When a child is in pain, you typically won't see smiles or laughter on their face. They may cry more easily and may not be comforted by breastfeeding or eating”

(Thelma, 33 years)

“In addition to crying, some children, particularly those aged five and older, are capable of expressing their pain verbally. It can be surprising to witness how effectively they communicate their pain, often more openly than some adults. When they experience pain, they tend to inform their parents, who, in turn, communicate it to the nurses. Children often mount more pressure on us when they are in pain than even adults”

(Ruth, 27 years)

#### 7.3.2. Sub theme 2: pain assessment guided by experience rather than routine process

At the hospital, pain assessment often diverges from routine procedures. Although basic vitals like pulse, temperature, and respiration are routinely checked upon admission, pain is not always systematically assessed unless a child is visibly crying or complaining. Instead, pain assessment is influenced by the patient's condition, such as cancer or fractures, and heavily relies on the nurse's experience. For instance, Matilda, a 25-year-old senior nurse, noted that with her 5 years of experience, she tends to assess pain more frequently.

“Pain assessment is not a routine practice on admission here, like we check the pulse, temperature and respiration on admission unless most of the time the child is crying or complains of pain. Assessment is dependent on the patient's condition like cancer or fracture as well as the level of experience, especially among senior nurses with over five years of experience, like a charge nurse I assess it more frequently”

Matilda (25 years old)

“The nurses here have some pain management tools pasted on the walls, including the faces pain scale and pain rating scale of 1 to 10. We the nurses hardly use it in the assessment because of time, workload and especially when the children are many”

(Joana, 28 years)

“Yes, I will say basically it's about skills and how long you’ve worked with children… I will say experience or exposure makes it easier for you to assess pain better than those who just came in”

Thelma (33 years old)

“Actually, after working for some time, you gain experience and realize that even though babies can't communicate verbally, you can still tell when they're in pain. As I mentioned earlier, their facial expressions, along with their reaction when you try to touch them, can indicate that there's pain somewhere”

Bridget (29 years old)

### 7.4. Theme 2: prioritizing pain management and barriers faced

This theme highlighted that although the nurses in this study prioritized effective pediatric pain management, they encountered several obstacles in ensuring its successful implementation.

#### 7.4.1. Subtheme 1: prioritizing effective pain management

The participants of this study prioritized effective pain management, choosing paracetamol in forms like suppositories, tablets, syrup, or IV based on pain severity. For severe pain, such as from fractures, the nurses start with IV paracetamol, morphine, or sedatives, then switch to more accessible forms as pain subsides.

“We, the nurses, typically administer pain relief to children using paracetamol in the form of suppositories, tablets, syrup, or intravenous routes. The choice depends on the severity of the pain. Among these, paracetamol suppositories are often preferred because they are readily accessible, less expensive, and easy to administer. We nurses are happy when the children are happy, so we do not take pain management lightly”

(Esi, 31 years)

“Other children, especially those with severe pain from conditions like fractures, are initially given IV paracetamol, morphine and other sedatives such as ketamine and lidocaine. Once the pain subsides, nurses may switch to suppositories, tablets, or syrup, depending on the nature of the pain”

(Mary, 33 years)

In the ER, where every moment counts, participants prioritized pain management by ensuring a swift pain management for children. Despite resource challenges, they actively address pain, swiftly updating doctors and using all available methods to bring relief and comfort.

“For ER (emergency room), the nurses are always on our toes and are always ready to manage pain in children. We the nurses do our best to attend to the families and their children even though at times the resources may be scarce”

(Desmond, 31 years)

“We (the nurses) do not delay pain management if the child or their mothers report pain, sometimes after our non-pharmacologic management, we inform the doctors in charge and send reminders to order prescriptions to help relief the pain”

(Bridget, 29 years)

“We (the nurses) do not joke with managing pain here, because these children always cry and their parents are not happy so we are always making sure that their pains are managed to prevent some of these problems that comes with it. I always feel happy when I have achieved this”

(Aku, 32 years)

#### 7.4.2. Subtheme 2: barriers to paediatric pain management (medication and assessment)

In efforts to prioritize pain management, some participants mentioned the challenges of administering suppository paracetamol to children. They noted delays caused by diaper changes and the frequent occurrence of defecation shortly after administration, which complicated effective pain management.

“I personally don't favour suppository paracetamol over the other forms. Children often soil their diapers, and you have to perform perineal care before administering it, which can cause delays when you have other children to attend to”

Bridget (29 years old)

“The issue with suppositories is that sometimes when you administer them, they might defecate. You know, with children, even if you ask them not to defecate right after inserting the medication, some of them still do it”

Justice (28 years old)

Another barrier to pain prioritization and management was assessment. The nurses identified that pain assessment was underpinned by clinical experience. Due to this they narrated that assessing pain in babies is a challenging task, as their cries can signal various needs—pain, hunger, or discomfort.

“To me, yes, it is kind of difficult assessing pain in babies. Babies cry at everything so how would you know if they are crying because of pain or not. That is what makes pain assessment in babies difficult”

Ruth (27 years old)

“With babies, I will say it's difficult because they don't say anything all you hear is crying but the crying could mean pain, could mean hunger or could mean I need a diaper change, so it's left with you the nurse to know what the crying means”

Anita (35 years old)

### 7.5. Theme 3: blending compassion, flexibility, and cultural practices

#### 7.5.1. Subtheme 1: integrating compassion and flexibility in pain management beyond protocols

During painful procedures, the nurses often used compassionate practices like allowing mothers to breastfeed, sucking on a gloved finger, or administering glucose solutions to soothe children. These methods, although not standardized protocols, significantly ease their discomfort compared to those who do not receive such care.

“Eermm, during painful procedures we sometimes allow breastfeeding mothers to breastfeed or give formula feeds to divert their attention from the pain. It is not a laid down protocol but it works when we use it. Even though some may still cry, it cannot be compared to those may not be given this therapy”

(Martin, 26 years)

“Pain is also controlled in some children by allowing them to suckle on a gloved finger in their mouth whilst the doctor is performing a painful procedure”

(Amina, 29 years)

Other nonpharmacologic management of pain recounted include cuddling the babies, putting liquid with glucose in the mouth or 10% dextrose, cold and warm compresses as followed:

“Babies are wrapped and cuddled sometimes to relieve them of pain. Also, drops of liquid containing sugar or 10% dextrose can also be given during a painful procedure. As they suckle on these things the procedure is performed to prevent them from the realisation of the pain. And after painful procedures, we ask their mothers to breastfeed them”

(Mary, 33 years)

“We normally use cold compresses when there is veinous infiltration or extravasation and becomes swollen. To manage these, we give the mothers ice cubes to put on it, or sometimes warm compresses, especially children with sickle cell who have swollen joints”

(Akosua, 34 years)

Other participants revealed the use of diversional therapy such as watching TV or playing a game on a phone, or encouraging them to join in with playtime:

“We let them watch television or play games with their mother’s phone. Also, we play with them so as to divert their attention from the pain they are experiencing. There is a playroom on the ward for them too. For the older children, their mothers give them gamepads too, and sometimes they watch cartoons on the TV, which helps in managing their pain”

Regina (27 years old)

#### 7.5.2. Subtheme 2: cultural resonance in pain relief: the impact of soothing traditions on crying

The nurses said that mothers in Ghana sing a range of lullabies to soothe their crying children, revealing the deep emotional impact of these songs.

“In Ghana, mothers have a variety of lullabies that they sing to soothe their crying children. It's fascinating to observe how these songs can effectively calm the children, and the crying often resumes once the singing stops”

Desmond (31 years old)

“Mothers also have a role to play in relieving babies of pain by cuddling them, breastfeeding them and placing them on their laps and playing with the babies and most importantly in Ghana, the mothers sometimes tie the babies and their backs with their clothes when they are in pain and crying and take strolls till these babies fall asleep”

(Aku, 32 years old)

#### 7.5.3. Subtheme 3: empowering families through pain management

The nurses recounted that before painful procedures, they ensured clear communication with parents and children about the process and pain management. According to them, this helped alleviate the distress of parents who might otherwise be overwhelmed by their child's suffering, highlighting the importance of explanation and reassurance in pain management.

“Whenever are about to perform any procedures that will cause pain, we make the parents and aware and also inform then the nature of the pain to be expected. If the child could also understand we inform the child too. However, we make them aware of why the procedure is done and ways we could reduce the pain being experienced”

(Ruth, 27 years)

“Sometimes when you are performing painful procedures on children, you will see their mothers' shedding tears or crying, so it is important to let the mothers know the importance of those procedures and be aware of the pain that comes with it and how it will be managed so that it will help mothers to overcome this. It comes down to explanation, reassurance and talking to the mothers because you sometimes see the mothers' shedding tears when they see their children crying uncontrollably during a painful procedure”

(Desmond, 31 years)

### 7.6. Theme 4: health care providers proactive strategies and sensitivity in paediatric pain management

This theme revealed that health care providers are proactive in assessing pediatric pain, demonstrating high sensitivity and a prompt response.

#### 7.6.1. Subtheme 1: clinician assessment and strategies in paediatric pain management

In this hospital, doctors play a crucial role in paediatric pain management, carefully assessing children before prescribing pain relief and occasionally offering comfort.

“Doctors also assess the children physically and physiologically prior to pain prescription, and from the outcome, they prescribe the right analgesics for them. Sometimes they also give instructions on the specific position to place the child so that the child does not experience pain”

Desmond (31 years old)

“In this hospital, the protocol is for doctors to set IV lines for us to administer pain medications where necessary. Even though it is not common to see doctors consoling patients regarding pains experienced, there are times you find some doctors do that. The doctors also have to increase the dosages and frequency of medications when pain is unrelieved, but sometimes we just have to report the outcome of the pain management to the doctors for them to know what to do next”

(Akosua, 34 years)

#### 7.6.2. Subtheme 2: high sensitivity and prompt response to pediatric pain by nurses

In the oncology unit, nurses are highly attuned to the children's pain, ensuring that prescribed medications are administered promptly. Their deep sensitivity to the children's suffering motivates them to act swiftly, providing comfort and relief without hesitation.

“This is an oncology unit, and we know the kind of conditions the children here come with, so the nurses on the ward are very sensitive to the children in pain. We administer the prescribed medication to relieve the children of their pain on time and as prescribed”

Gideon (31 years old)

“We are very sensitive to children's pain; how can you sit as a nurse when a child is crying because of pain? We quickly assist mothers to manage the pain when it occurs. We cannot see a child screaming and crying, and you sit down without doing anything. I can rate the sensitivity as 90%”

Akosua (34 years old)

## 8. Discussions

### 8.1. Pain management practices among pediatric nurses

The current study explored pain management techniques among urban teaching hospital pediatric nurses. The study identified crying, facial expressions, and verbalization as primary indicators for assessing pain in children. However, interpreting these signals, especially crying, can be challenging for new nurses since it may signify various issues. Therefore, specific training in pain identification and management is crucial for pediatric nurses. Such training can alleviate emotional and physical distress for both patients and their parents and is essential for initiating effective clinical approaches to pain management, which is particularly important in developing countries where health care resources may be limited.^20^

A significant finding of the study was the effective use of nonpharmacological methods by nurses to manage pediatric pain during procedures. Techniques such as allowing mothers to breastfeed, encouraging children to suck on fingers, administering 10% dextrose, and singing Ghanaian lullabies were found to be cost-effective, efficient, and easily accessible. Previous studies corroborate these findings by endorsing the use of breastmilk or sucrose for pain relief in neonates and infants during procedures.^15,23^ These methods should be integrated into pain management practices to complement pharmacological treatments. For developing countries like Ghana, where access to advanced medications and technologies may be restricted, adopting these nonpharmacological strategies can enhance pain management and improve patient care. Implementing these techniques, supported by appropriate training, can address local needs, reduce health care costs, and offer culturally sensitive care, ultimately contributing to better health outcomes and reduced caregiver burden in resource-constrained settings and developed countries.

Informing parents about painful procedures in advance, providing reassurance, and involving them in pain management techniques were found to be effective strategies in pediatric pain management, particularly for positioning and pain reporting. Since pain is subjective and parents often use methods^16^ they find effective, actively involving them can help identify successful strategies and train them to manage pain more effectively, even after discharge. This approach not only improves immediate pain management but also empowers parents to continue providing effective care at home. For developing countries, including Ghana, these findings highlight the importance of incorporating parent education and involvement in pain management protocols.

The assessment of nurses' attitudes revealed positive views towards pain management, with nurses being attentive to the pain-related needs of children and intervening as necessary. This responsiveness is vital, as unmanaged pain can significantly affect the emotional, social, and overall health of children and their parents, leading to potential noncompliance and increased stress for health care providers. Encouraging prompt and positive attention to pediatric pain is essential, and this observation is consistent with similar findings in district hospitals in Ghana.^16^ For Ghana and other developing countries, fostering positive attitudes among nurses and supporting them with adequate training and resources are crucial for improving pediatric pain management. This approach can enhance the quality of care, address the challenges of limited resources, and contribute to better health outcomes for children and their families.

Nurses recognized the unique challenges of managing pediatric pain compared to adult pain, underscoring the need for increased motivation and support. To address these challenges effectively, it is crucial to provide adequate resources and implement supportive policies that promote a multidisciplinary approach. Facilitating collaboration among health care providers is essential for optimal pediatric pain management, as emphasized by previous studies.^6^ For developing countries, including Ghana, these findings highlight the importance of investing in comprehensive support systems for nurses, such as access to training, resources, and collaborative networks. By enhancing these aspects, health care systems in these regions can improve the quality of pediatric pain management, address resource limitations, and ultimately provide better care for children in need.

### 8.2. Limitation

Although this study offers valuable insights and may have similar applicability in other African countries, findings have limited generalizability in industrialized areas and may not fully capture the variations and nuances in pain management practices that could exist among a larger and more diverse group of nurses.

### 8.3. Implications for future research studies

Future research should focus on evaluating the effectiveness of nonpharmacological pain management strategies in diverse settings, including resource-limited environments. In addition, studies should explore the impact of parent involvement in pain management on long-term outcomes and the development of training programs for nurses in developing countries to enhance their skills and support in pediatric pain management. Furthermore, comforting and culturally sensitive care strategies are, and should continue to be, applicable worldwide.

## 9. Conclusion

In conclusion, this study sheds light on the multifaceted approaches used by pediatric nurses to manage pain in children. Both pharmacological and nonpharmacological methods play crucial roles in pediatric pain management, with paracetamol (acetaminophen) emerging as the primary analgesic. Moreover, the nurses in this study had a positive attitude towards pediatric pain management to enhance comfort.

## Disclosures

The authors have no conflict of interest to declare.
